# Cu(II)-based complex loaded with drug paclitaxel hydrogels against thyroid cancer and optimizing novel derivatives

**DOI:** 10.1038/s41598-024-63940-w

**Published:** 2024-06-06

**Authors:** Hui-Hui Wang, He-Liang Yin, Wei-Wei Yin, Yong-Li Song, Hong Chen

**Affiliations:** 1 Department of Endocrinology, Qiqihar First Hospital, Qiqihar, Heilongjiang, China; 2 Department of Integrative Chinese and Western Medicine, School of Traditional Chinese Medicine, Southern Medical University, Guangzhou, Guangdong China; 3 Department of General Surgery, Qiqihar First Hospital, Qiqihar, Heilongjiang, China; 4 Department of Science and Education, Qiqihar First Hospital, Qiqihar, Heilongjiang, China; 5 Department of Oncology, Heilongjiang Academy of Chinese Medicine, Harbin, Heilongjiang, China

**Keywords:** Coordination polymers, Learning simulation, Hydrogels, thyroid cancer, Biochemistry, Chemical biology, Molecular biology, Structural biology

## Abstract

This study introduces a novel approach for synthesizing a Cu(II)-based coordination polymer (CP), {[Cu(L)(4,4´-OBA)]·H_2_O}n (**1**), using a mixed ligand method. The CP was successfully prepared by reacting Cu(NO_3_)_2_·3H_2_O with the ligand 3,6-bis(benzimidazol-1-yl)pyridazine in the presence of 4,4´-H_2_OBA, demonstrating an innovative synthesis strategy. Furthermore, a novel hydrogel composed of hyaluronic acid (HA) and carboxymethyl chitosan (CMCS) with a porous structure was developed for drug delivery purposes. This hydrogel facilitates the encapsulation of CP**1**, and enables the loading of paclitaxel onto the composite to form HA/CMCS-CP**1**@paclitaxel. In vitro cell experiments demonstrated the promising modulation of thyroid cancer biomarker genes S100A6 and ARID1A by HA/CMCS-CP**1**@paclitaxel. Finally, reinforcement learning simulations were employed to optimize novel metal–organic frameworks, underscoring the innovative contributions of this study.

## Introduction

Thyroid cancer (TC) is a common endocrine cancer mainly originating from follicular epithelial cells, and is most common in middle-aged and elderly women. It is divided into papillary thyroid carcinoma (PTC), follicular thyroid carcinoma (FTC), poorly differentiated thyroid carcinoma (PDTC), and metaplastic thyroid carcinoma (ATC), of which PTC and FTC account for 95% of thyroid cancer cases^1^. PTC and FTC account for 95% of thyroid cancer cases. ATC is very rare, accounting for about 2% of thyroid malignancies. The incidence of thyroid cancer is increasing. Surgical resection is currently the standard treatment for most thyroid cancer patients. However, the poor survival rate of patients with advanced thyroid cancer suggests that there is still a lack of effective treatment strategies^2^. S100A6 is a member of the S100 family of calcium-binding proteins. S100A6 has been shown to promote epithelial-mesenchymal transition, proliferation, and migration of various cancer cells. The ARID subunit family is thought to enhance the activity of the SWI/SNF complex by recruiting the ATPase catalytic module, and among them, ARID1A is the most frequently mutated gene in the SWI/SNF subunit^3^. ARID1A mRNA expression is negatively correlated with the degree of malignancy of differentiation in thyroid cancer. ARID1A mRNA expression is negatively correlated with the degree of malignant differentiation of thyroid cancer.

**Scheme 1:**
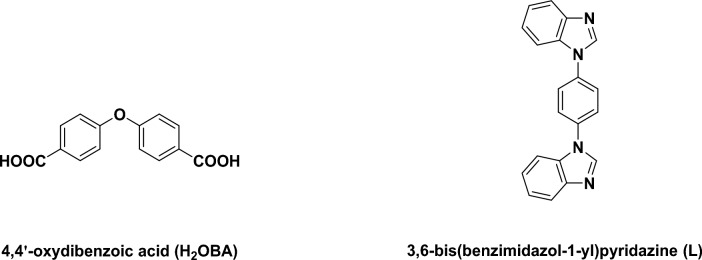
Chemical diagrams of the ligands utilized in this study.

The last decades have seen a tremendous growth in coordination polymer (CP) fabrication and design because of their attractive structure characteristics and potentially beneficial uses, like gas adsorption and separation, luminescence, catalysis, sensors, and biomedicine^4–7^. Remarkably, the controllable assembling of coordination polymers having specific configurations and properties remains a major challenge fraught with difficuities because of the unpredictable nature of the generated complexes in regulating the chemical circumstances (e.g., metal ions, organic ligands, reagent ratios, solvents, pH levels, temperatures, etc.)^8–11^. The use of N-donor ligands for the construction of CPs has been a long-standing research topic among chemists, and numerous N-donor ligands were covered and investigated so far. Much attention has been devoted to the study of ligands derived from azole heterocycles, having favorable coordination capacity and diversified coordination patterns. Polydentate nitrogen heterocyclic ligands (azoles) containing a five-membered ring are widely utilized for the building of supramolecular architectures^12–14^. In particular, the organic ligands triazole, imidazole and benzimidazole played a significant part in the engineering and structuring of CPs^15–17^. Motivated by such thoughts, we chose 3,6-bis(imidazol-1-yl)pyridazine (L) as the main ligand because its invariant structural units are favorable for tuning the coordination pattern of the secondary ligand. In current study, we performed the solvothermal reaction of Cu(NO_3_)_2_·3H_2_O with L in the existence of a V-shape carboxylic acid co-ligand 4,4´-H_2_OBA (Scheme 1) to produce a novel mixed-ligand Cu(II) coordination polymer {[Cu(L)(4,4´-BOA)]·H_2_O}_n_ (**1**).

As an anti-microtubule chemotherapeutic drug, paclitaxel can inhibit the growth of thyroid cancer cells by interfering with the formation and deaggregation of microtubulin, resulting in the stop of cell division^18–20^. Traditional drug delivery methods, such as oral or local injection, often lead to short drug action time in the body, difficult to maintain a stable blood concentration, thus affecting the therapeutic effect^21^. As a drug carrier, the unique three-dimensional porous network structure of hydrogel enables the drug to be evenly distributed in it, and the drug effect can be sustained at the lesion site through slow release^22,23^. This continuous release property can not only extend the duration of action of the drug, but also reduce the number of dosing times, thus reducing the burden on patients. As natural polysaccharides, hyaluronic acid and carboxymethyl chitosan have good biocompatibility and biodegradability, and have been widely used in biomedicine, drug controlled release, tissue engineering and other fields^24,25^.

In this study, we innovatively synthesized metal gel particles loaded with paclitaxel and assessed their impact on thyroid cancer cells. Treatment of CAL-62 cells with varying concentrations of these particles resulted in a significant decrease in thyroid cancer cell activity, correlating with increased drug concentration. Notably, the expression levels of S100A6 and ARID1A, key marker genes for thyroid cancer, were markedly downregulated and upregulated, respectively. These findings suggest that metal gel@Paclitaxel effectively suppressed thyroid cancer cell proliferation by modulating the expression of S100A6 and ARID1A.

Moreover, the conventional approach to designing and developing novel drug molecules is hindered by its complexity, time-consuming nature, and high cost. To overcome these challenges, we employed reinforcement learning methods, known for their ability to conduct high-throughput evaluations in a short timeframe, to optimize potential drug structures. Additionally, molecular docking simulations were performed on the synthesized and optimized structures to elucidate the underlying mechanisms of their biological activities.

## Experimental

### Chemicals and measurements

Cu (NO_3_)_2_·3H_2_O used in the experiment HBF_4_、 Methanol and CH_3_CN were purchased from Shanghai Aladdin Biochemical Technology Co., Ltd. Organic ligands 3,6-bis (benzimidazol-1-yl) pyridine (L) and 4,4 '- oxydibenzoid acid (H_2_OBA), HA powder, CMCS powder, paclitaxel, and calf serum were purchased from Shanghai McLean Biochemical Technology Co., Ltd. Distilled water is prepared from laboratory pure water mechanism. All experimental reagents are for direct use and have not been further purified. Scanning electron microscopy (SEM; Hitachi S-4800) was employed to examine the morphology and size of the samples in both aqueous and organic phases. X-ray diffraction (XRD) analysis of dried samples from the aqueous phase was conducted using Cu Kα radiation (λ = 1.54056 Å) in the 2*θ* range of 10° to 80°. Fourier-transform infrared spectroscopy (FTIR) was carried out using an IFS 66 V/S instrument (Bruker) across the range of 500–4000 cm^−1^.

### Preparation and characterization for {[Cu(L)(4,4´-*BOA*)]·H_2_O}_n_ (1)

A solution of Cu(NO_3_)_2_·3H_2_O (0.100 mmol, 24.0 mg), L (0.100 mmol, 31.2 mg), 4,4´ -H_2_OBA (0.100 mmol, 21.4 mg), HBF_4_ (8 drops, 37% aq) in 6 mL of solution (V_H2O_:V_CH3CN_ = 1:1) were sealed in a 25 mL Teflon-lined autoclave, and warmed at 160℃ for overnight, and next slowly cooled to ambinet condition. Blue crystals of 1 were gathered and cleaned with water and methanol. Yield: 29 mg (~ 45% according to L). Elemental analysis (%): Anal. Calcd for C_32_H_20_N_6_O_5_Cu: H, 3.19; C, 60.81; N, 13.30. Found: H, 3.18; C, 60.63; N, 13.26. IR (cm^−1^, KBr pellets): ν (O − H, stretching vibrations) = 3387 s, br, ν asym (− CO_2_^−^) = 1619 s (1589 s, 1652w), ν sym (− CO_2_^−^) = 1417 m, 1360 s(1436 s), 1393 s, ν (aromatic ring stretching) = 1480 m (1480w), ν (C − H out-of-plane deformation vibrations, substituted aromatics) = 763 m, 879w, 833 m, 696w (869 s, 824 m, 652 m), ν (C − H in-plane deformation vibrations, substituted aromatics) = 1181w, 1281w, 1038 m (1160 m, 1145 m, 1114w).

**1**’s single crystal data were acquired utilizing a Mercury CCD diffractometer controlled by computer at RT with 0.71073 Å graphite monochromated Mo–K*α* radiation. The solution and refinement of the structures is achieved by the direct method ShelXS structural solution procedure and the ShelXL refinement package applying the least squares method ^26^. There lattice water molecules per unit cell were extruded *through* PLATON program^27^. **1**’s crystallographic data are listed in Table 1.

**Table 1:** Refinement specifications and crystallographic properties of CP1.

Empirical formula	C_32_H_20_CuN_6_O_5_
Formula weight	632.08
Temperature/K	293(2)
Crystal system	triclinic
Space group	*P* $\overline{1 }$
a/Å	10.2631(2)
b/Å	10.95630(13)
c/Å	12.1458(5)
α/°	104.629(2)
β/°	93.5524(14)
γ/°	103.6752(19)
Volume/Å^3^	1273.20(6)
Z	2
ρ_calc_g/cm^3^	1.649
μ/mm^−1^	0.917
Data/restraints/parameters	5202/0/397
Goodness-of-fit on F^2^	1.049
Final R indexes [I > = 2σ (I)]	R_1_ = 0.0326, ωR_2_ = 0.0649
Final R indexes [all data]	R_1_ = 0.0383, ωR_2_ = 0.0679
Largest diff. peak/hole/e Å^−3^	0.29/− 0.30

### Preparation of HA/CMCS hydrogel

First, 1 g HA powder and 2 g CMCS powder (provided by Sinopod Chemical Reagents Co., LTD) were weighed and dissolved in deionized water, respectively, to obtain a concentration of 2 wt% HA solution and 4 wt% CMCS solution. Then, at room temperature, the 1-Ethyl-3-(3-dimethylaminopropyl) carbodiimide / *N*-Hydroxysuccinimide(EDC/NHS) solution was slowly dripped into the HA solution and stirred continuously for 20 min to fully activate the carboxyl groups in the HA molecules. After that, the prepared HA mixture and CMCS solution were quickly added to the mold according to the volume ratio of 1:1, and the mixture was quickly stirred to ensure uniform mixing. After the gelation reaction was completed, deionized water was used to rinse several times to completely remove the unreacted raw materials and by-products, so as to obtain pure HA/CMCS hydrogel.

### Preparation of paclitaxel-loaded metal gel

Among them, the Cu coordination polymer was placed in 10 mg/ml paclitaxel solution and sonicated for 30 min to fully adsorb paclitaxel drug molecules, and then mixed with CMCS solution to prepare paclitaxel loaded metal gel. The sample's microscopic morphology was observed using a scanning electron microscope. Prior to the test, the sample underwent freeze-drying and gold spraying.

### CCK-8 assay

CAL-62 cells were purchased from ATCC and cultured in a saturated humidity constant cell culture incubator at 37 ℃ with 5% CO_2_ using DMEM medium containing 10% fetal bovine serum (FBS). The logarithmic growth phase cells were collected and inoculated in 96-well cell culture plates, and different concentrations of system were added for 48 h. 20 μl of CCK-8 reagent was added, and the cells were incubated for 15 min with gentle shaking and incubation under light protection, and the absorbance (OD) values of the cells were detected at 450 nm.

### Real-time Polymerase Chain Reaction (Realtime PCR)

The total RNA was isolated from CAL-62 cells using Trizol reagent (Invitrogen, USA). The cDNA was synthesized by SuperScript IV cDNA Kit (Invitrogen, USA) according to the manufacturer's instructions. The relative mRNA levels of S100A6 and ARID1A was determined by PowerUp SYBR Green kit (Invitrogen, USA) on the ABI 7500 Fast Realtime PCR system (Applied Biosystem, USA). The relative mRNA expression was calculated by 2^–∆∆Ct^ method using GAPDH as internal control.

### Simulation details

It has been demonstrated that the complex which has inhibition effect on the aggregation procedure of amyloid-β (Aβ) will show biological activity^28^, in addition, it has been proven that if the given drug molecule exhibits significant inhibitory activity on Aβ, it is expected to suppress accumulation of certain other amyloid proteins, therefore, when using Aβ as the receptor for qualifying the biological activity, the probed drug molecule would potentially has broader biological activity towards other types of proteins. Thus, we obtained the crystal structures of the Aβ protein (4TVK) downloaded from the Protein Data Bank as a receiver for the molecular docking simulations. The preparation steps for the molecular docking simulation were completed using AutoDockTools (1.5.7), the number of the grid points for the docking box was 100, and the center of mass of 4TVK was used as the center of the grid box. The Gasteiger charges were assigned, and the Lamarckian Genetic Algorithm (LGA) was used for sampling the binding poses. Molecular docking simulations were conducted using AutoDock 4 (4.2).

The molecule deep Q-networks (MolDQN) algorithm^29^ was used for conducting the reinforcement learning simulation, the more detailed implementations can be found in the original paper. Reinforcement learning rewards include a binding energy between the complex and the receptor protein 4TVK, a synthetic availability (SA) score characterizing the ease of synthesis of the optimized structure, and a quantitative estimation of drug similarity (QED) score characterizing the degree of similarity of the optimized structure to known drug molecules. The binding energy was estimated by QuickVina 2 (2.1), the SA score was calculated by the GYM-molecule tool, and the QED score was calculated by RDKit (https://www.rdkit.org). During the reinforcement learning simulation, Open Babel (2.4) generated and optimized 6500 structures, with each generated structure allowing 16 revisions to the model architecture.

## Results and discussion

### Crystal structure description

**Figure 1:**
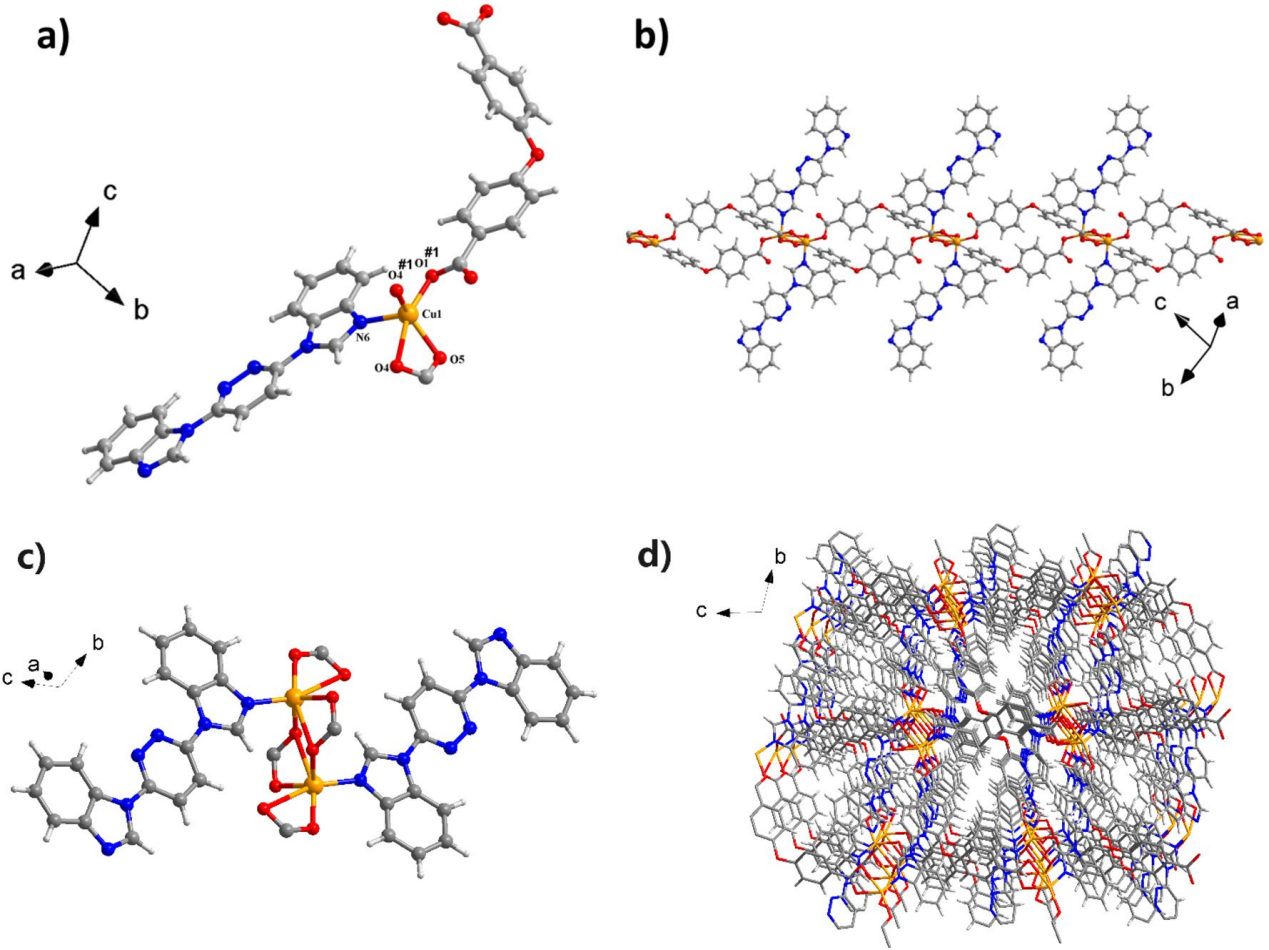
(**a**) Asymmetrical view in units of 1 (symmetrical code #1: + X,1 + Y,1 + Z). (**b**) The 1D Cu-ligands chains in CP**1**. (**c**) The coordination mode of ligand L in CP**1**. (d) The 3D stacking structure of CP**1**.

X-ray crystal experiments indicate that CP**1** crystallizes in the monoclinic *P* $\overline{1 }$ space group, and have a 1D chains configuration. The unsymmetrical units consist of an individual Cu^II^ ion, an L ligand and a 4,4´-OBA ligand as well as a lattice H_2_O molecule. As illustrated in Fig. 1a, the Cu1 ion was situated in a Trigonal bipyramid geometry with a τ value of 3.18 as calculated via the SHAPE software by one nitrogen (N6) donor centers of L ligand and four oxygen (O5^#2^, O4^#2^, O4^#1^, and O1) centers from three 4,4´-OBA ligands^30^. The lengths of Cu–O bond are in the range of 1.9442(14)–2.4607(15) Å and lengths of Cu − N bond is 2.0087(16) Å, which are equivalent to the results observed in alternative Cu(II)-based coordination polymers structured from mixed ligand systems^31–33^. The Cu1 atom is located at the core of the pentahedron and the ligand atoms are located at the vertices of the pentahedron. Two adjacent Cu1 centers are connected by two μ2-1,3-carboxylic acid O atoms in two different 4,4´-OBA ligands to form a binuclear [Cu2(CO2)2N2] building block with a Cu-Cu distance of 3.490 Å. These trinuclear units are subsequently interconnected by additional 4,4´-OBA ligands in a (κ^1^)-(κ^1^-κ^2^)-μ3 coordination mode, forming a 1D chain configuration (Fig. 1b), whereas the L ligands adopt a monodentate bridge coordination mode (Fig. 1c). which form a one-dimensional chain structure (Fig. 1b), while the L ligand adopts a monodentate bridge coordination mode. The geometric configuration of the five-coordinated transition metal Cu^2+^ in CP**1** is similar to those in reported related complexes,such as: [Cu(dppn)_2_(ClO_4_)]ClO_4_^34^ and {[Cu_4_(L)_2_(1,3-HBDC)(1,3-BDC)(μ_3_-OH)_4_]·ClO_4_·2.6H_2_O}_n_ and {[Cu_2_L(2,6-PYDC)_2_(H_2_O)_2_]·4.4H_2_O}^35^. Although the specific coordination N and/or O atoms are different, the bond valence of the metal cation Cu is the same, both of them are + 2 valence states. In addition, in the reported known complexes [Mn(L)(2,5-TDC)(H_2_O)]_n_, [Mn(L)(2,6-PYDC)(H_2_O)]_n_, [Mn(L)(1,4-NDC)]_n_^36^ and {[Cu(L)(HCOO)_2_]·H_2_O·CH_3_OH}_n_ (L = 3,6-bis(iminazol-1-yl) pyridine)^37^, each metal cation is associated with one N atom from the ligand 3,6-bis (iminazol-1-yl) pyridine. The Cu–N and Cu–O bond lengths/angles of CP**1** are similar to those in the above mentioned complexes and fall within their normal ranges. The adjacent 1D chain extends into the 2D layered configuration further through interaction of pyridazine ring of the L ligand and benzene ring of the OBA ligand with a center distance of 3.553(2) Å. Figure 1d depicts the final 3D stacking structure.

**Figure 2:**
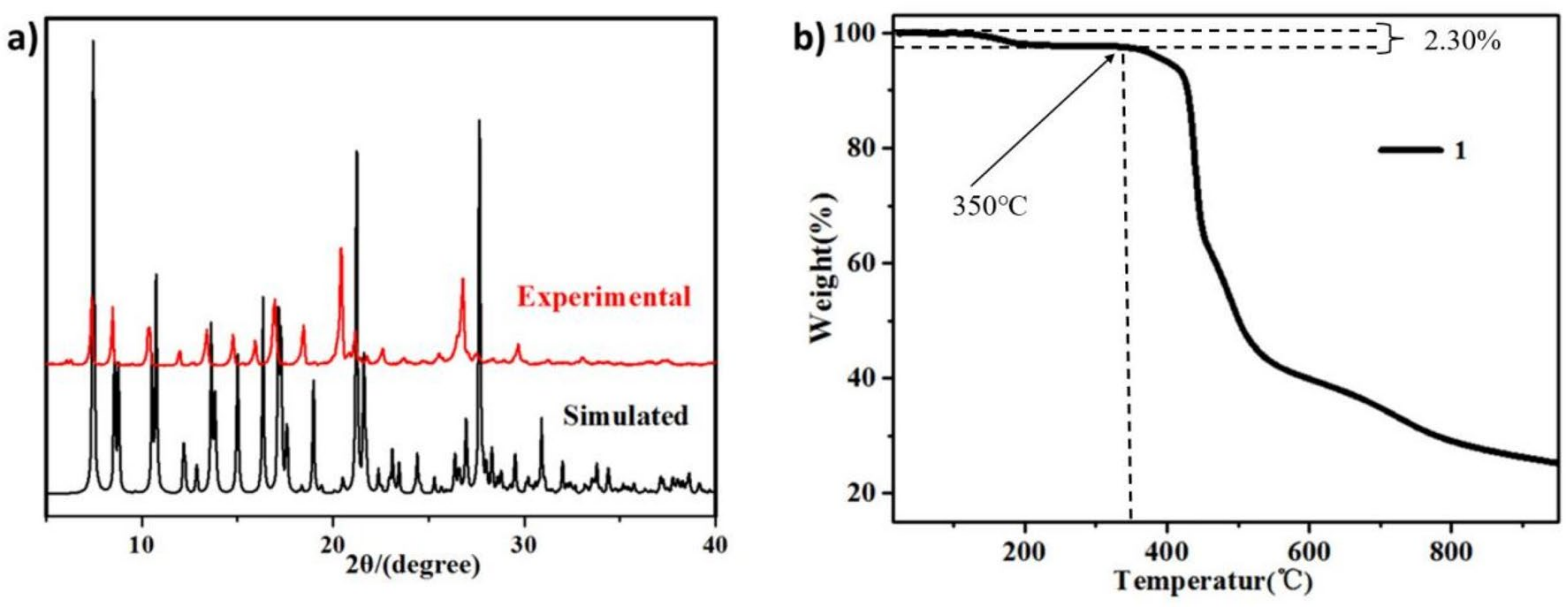
(**a**) The PXRD result of CP**1**. (**b**) The TGA data for CP**1**.

As shown in Fig. 2a, by comparing the experimental and simulated PXRD plots of CP**1**, we find that the simulated diffraction peak and the experimentally diffracted peaks match very well at the critical position, which indicates that the phase purity of the sample is very high. The difference in intensities could be due to the preferential orientations of the crystalline sample. Specifically, intense and typical diffraction peaks were observed at 2*θ* = 5.7°, 8.4°, 11.3°, 20.4°, and 26.4°, confirming the crystalline structure of CP**1**. The thermal stabilities of CP**1** were measured over the 25–1000 °C range (Fig. 2b) at a temperature rise rate of 5 °C min-1 in an argon gas stream. The decrease in weight loss of CP**1** was 2.3% in the scale of 25 to 186 °C, which could be related to removing of a lattice water molecule. CP**1** is thermally unstable up to 350 °C, beyond which the framework starts to collapse.

### Micromorphology of the hydrogels

**Figure 3:**
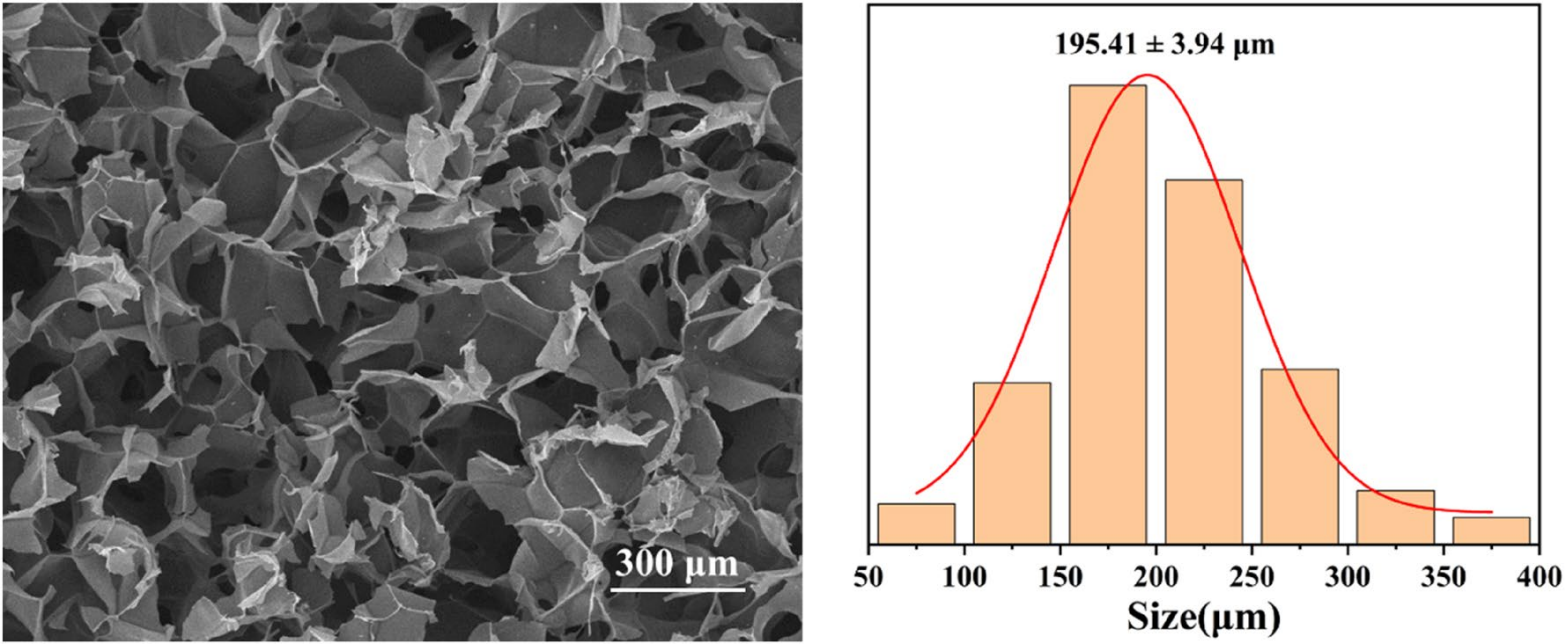
Microstructure and pore size distribution of HA/CMCS hydrogels.

Hydrogels are formed by physical or chemical cross-linking of hydrophilic polymers and contain a large number of interconnected tiny pores, which not only provide sufficient loading space for the drug, but also ensure the smooth flow of the drug during release. Based on the chemical synthesis method, we successfully prepared HA/CMCS hydrogels and observed their internal micromorphology by scanning electron microscopy. As shown in Fig. 3, the inside of the freeze-dried HA/CMCS gel showed a clear three-dimensional porous structure. These pores were highly connected, and the pore size was mainly concentrated in the range of 195.41 ± 3.94 μm, which is essential for uniform adsorption and slow release of drugs. Therefore, HA/CMCS hydrogel is considered as an excellent drug carrier due to its unique structure and excellent properties, and is expected to play an important role in drug controlled release system.

### In Vitro cytotoxicity of HA/CMCS-CP1@Paclitaxel

**Figure 4:**
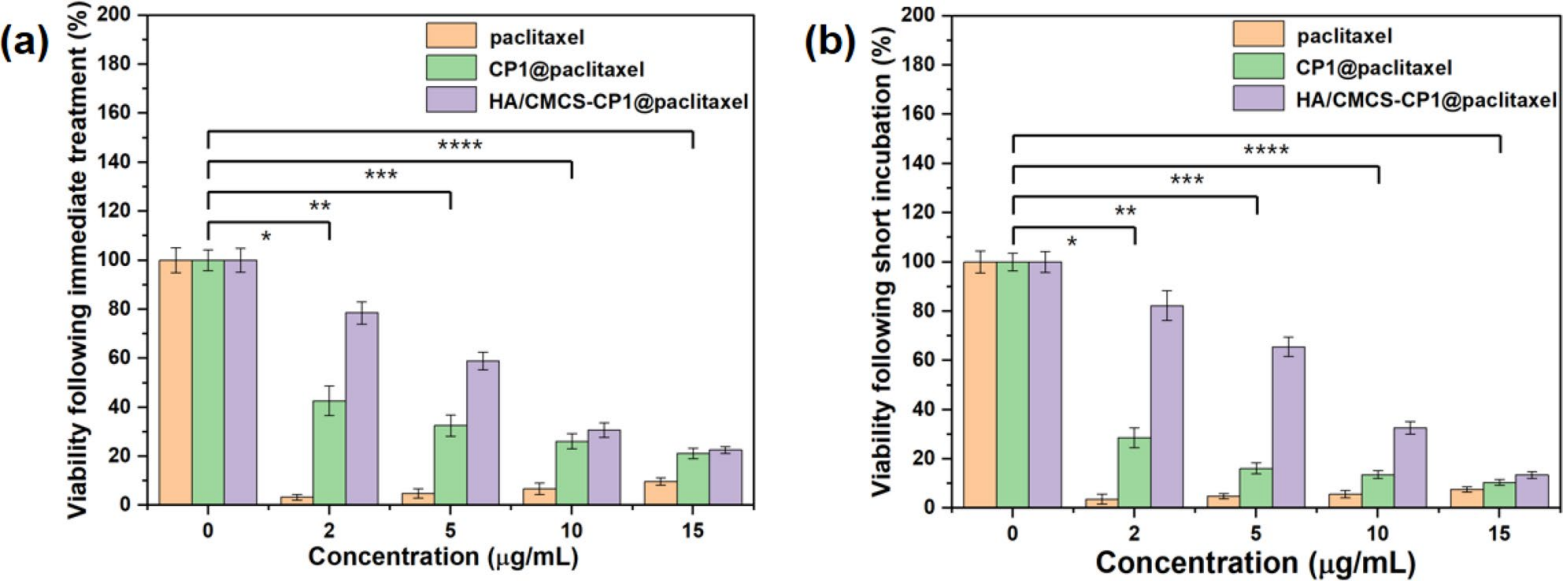
After 72 h of treatment with paclitaxel, CP1@paclitaxel, and HA/CMCS-CP1@paclitaxel, the viability of HeLa cells was assessed using the MTS assay. (**a**) Freshly prepared stock solutions. (**b**) Stock solutions stored at room temperature for 4 h (n = 4); ***P ≤ 0.0001, **P ≤ 0.001, *P ≤ 0.01, *P* ≤ 0.05).

To compare the cytotoxicity of HA/CMCS-CP**1**@paclitaxel, we evaluated the anticancer effects of free paclitaxel, CP**1**@paclitaxel, and HA/CMCS-CP**1**@paclitaxel using MTS assays. Cell behavior was observed through automated image acquisition and analysis. For each experiment, stock solutions of free paclitaxel, CP**1**@paclitaxel, and HA/CMCS-CP**1**@paclitaxel were prepared at the same concentration (1 mg/mL). These solutions were used immediately or incubated at room temperature for 4 h before being applied to cell cultures. Cell viability was measured after 48 h using the MTS assay. The results showed that at a concentration of 2 μg/mL, HeLa cells treated with free paclitaxel exhibited significant toxicity within 48 h, whereas CP**1**@paclitaxel and HA/CMCS-CP**1**@paclitaxel showed lower toxicity, with a concentration-dependent trend. CP**1**@paclitaxel was more toxic than HA/CMCS-CP**1**@paclitaxel (Fig. 4a). This indicates that HA/CMCS provides a protective effect on the encapsulated drug, preventing burst release. Furthermore, when using the CP**1**@paclitaxel stock solution 4 h after preparation, its cytotoxicity at lower concentrations was more pronounced compared to immediate use, while HA/CMCS-CP**1**@paclitaxel did not show significant toxicity changes after short-term incubation (Fig. 4b). These results suggest that HA/CMCS-CP**1**@paclitaxel can slowly release paclitaxel at high drug concentrations, highlighting its potential as a controlled-release drug delivery carrier.

### HA/CMCS-CP1@Paclitaxel significantly suppresses cellular activity of thyroid *cancer* cells

**Figure 5:**
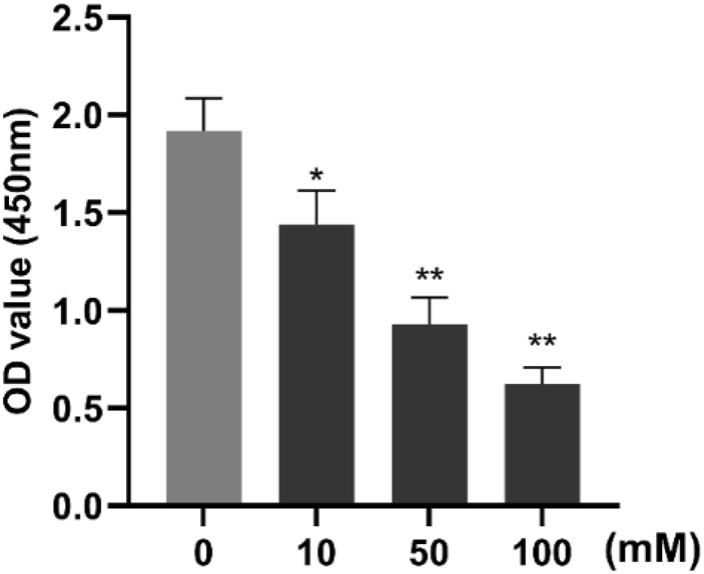
HA/CMCS-CP**1**@Paclitaxel significantly suppresses cellular activity of thyroid cancer cells. CAL-62 cells were treated with different concentrations of HA/CMCS-CP**1**@Paclitaxel for 48 h. Cell viability was determined by CCK-8 assay. * indicates *P* < 0.05, ** indicates *P* < 0.01.

In order to verify whether the HA/CMCS-CP**1**@Paclitaxel could inhibit the activity of thyroid cancer cells, the thyroid cancer cells CAL-62 were treated with different concentrations of the system for 48 h, and the cell activity was detected by CCK-8. As shown in Fig. 5, the HA/CMCS-CP**1**@Paclitaxel could significantly inhibit the cell activity of CAL-62 cells, and this inhibitory tendency showed a drug-dose dependence, the higher the concentration of HA/CMCS-CP**1**@Paclitaxel, the more the cell activity decreased. Our results suggest that HA/CMCS-CP**1**@Paclitaxel significantly inhibit the proliferation of thyroid cancer cells.

### HA/CMCS-CP1@Paclitaxel regulates the expression of S100A6 and ARID1A in thyroid *cancer* cells

**Figure 6:**
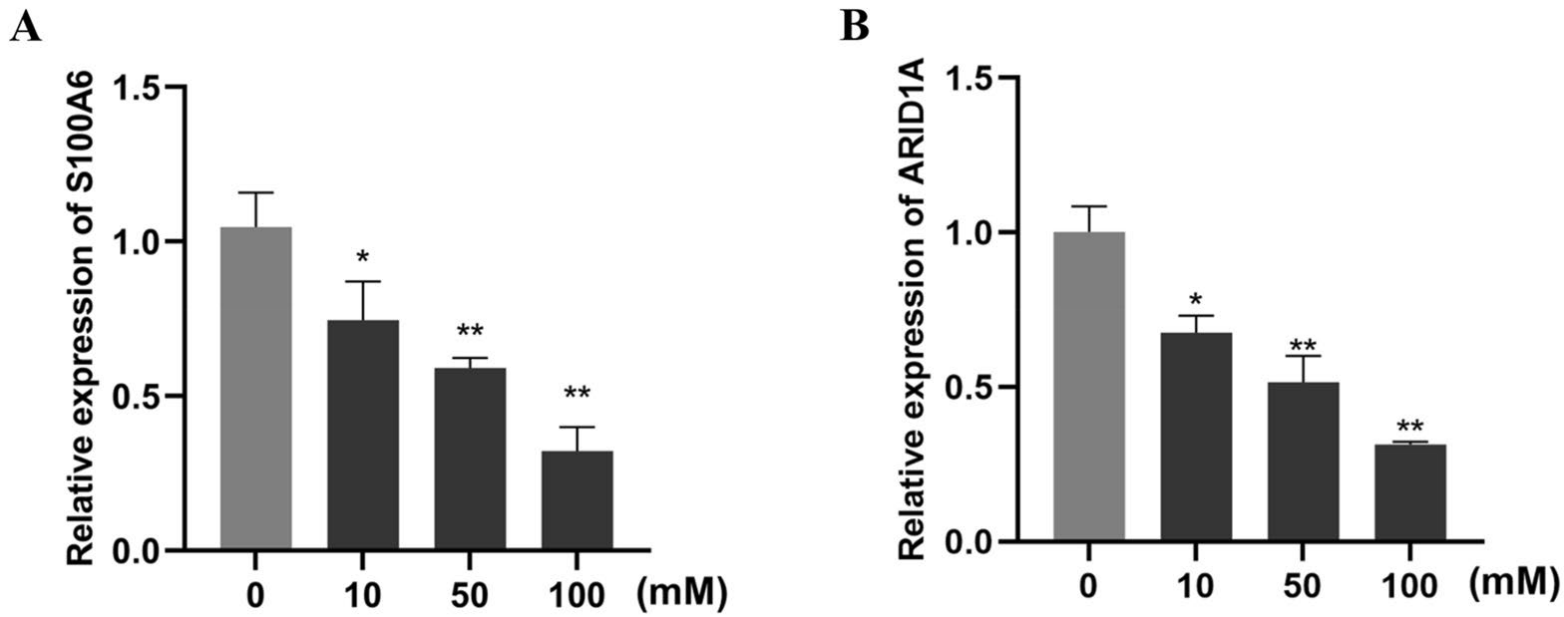
HA/CMCS-CP**1**@Paclitaxel regulates the relative expression of S100A6 and ARID1A in CAL-62 cells. CAL-62 cells were treated with different concentrations of the system for 48 h and the relative mRNA levels of S100A6 (**A**) and ARID1A (**B**) was measured using Realtime PCR. * indicates *P* < 0.05, ** indicateds *P* < 0.01.

In order to verify the regulatory mechanism of HA/CMCS-CP**1**@Paclitaxel on thyroid cancer cells, we treated CAL-62 cells with different concentrations of the system for 48 h. The relative expression of thyroid cancer marker genes S100A6 and ARID1A was detected using Realtime PCR. As shown in Fig. 6, the HA/CMCS-CP**1**@Paclitaxel could significantly reduce the expression of S100A6 in CAL-62 cells, and at the same time could up-regulate the mRNA level of ARID1A. These two regulatory trends correlated with the drug dose in a dose-dependent manner. Our results suggest that HA/CMCS-CP**1**@Paclitaxel inhibit the growth of thyroid cancer cells by down-regulating S100A6 and up-regulating ARID1A expression.

### Reinforced learning predictions and molecular docking simulations

**Figure 7:**
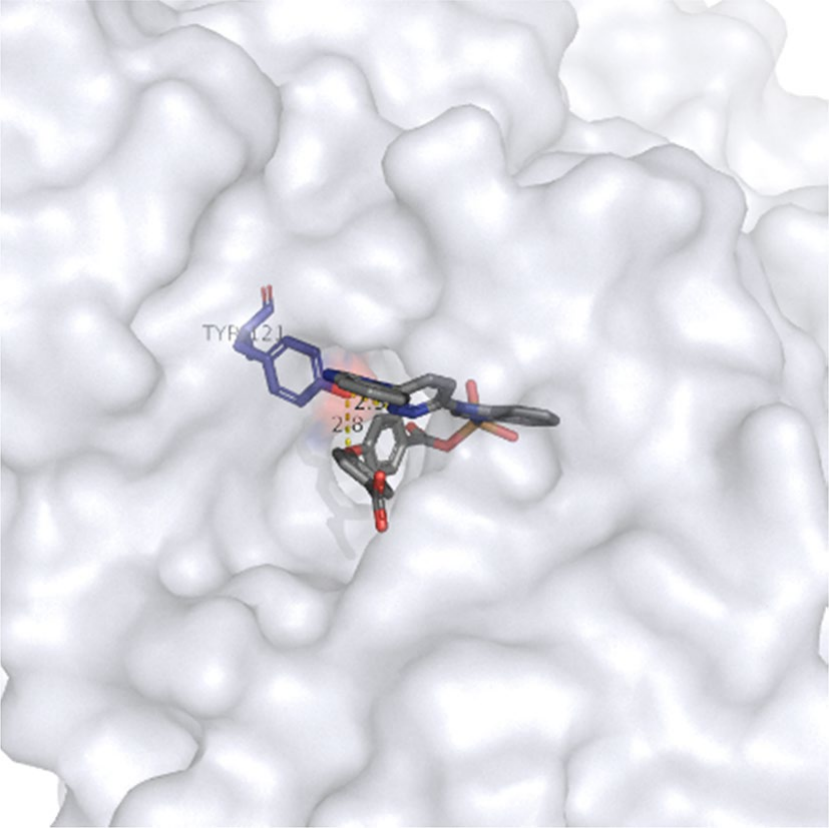
The docking pose between Cu complex and Aβ receptor (4TVK), two interactions were formed with active site from residue TYR-121. The binding energy was -11.9 kcal/mol, the inhibition constant was 1.96 nM.

Amyloid-β (Aβ) aggregation is shown to have an important function in the development of serious diseases, thus, any complexes that could exhibit inhibition effect on the aggregation process of Aβ will show biological activity. To learn the intrinsic mechanisms of the inhibitory effects of Cu complexes, we conducted molecular docking simulations to explore the interactions of Cu complexes with Aβ receptors, and the docking poses are shown in Fig. 7. The estimated binding energy and inhibition constant were -11.8 kcal/mol and 1.96 nM. Two binding interactions can be caught by the eye, both pyrimidine and ether groups were interacting with the phenolic hydroxyl group on residue TYR-121, and the hydrogen bond lengths were 2.3 and 2.8 Å. The findings above suggest that both moieties on the Cu complex have the activity to be involved into the interactions with receptor, therefore the biological activity has been revealed. To further explore possible structures based on the carboxyl rich moiety for biological activity, the reinforcement learning simulation has been performed and 6500 generated structures were examined.

**Figure 8:**
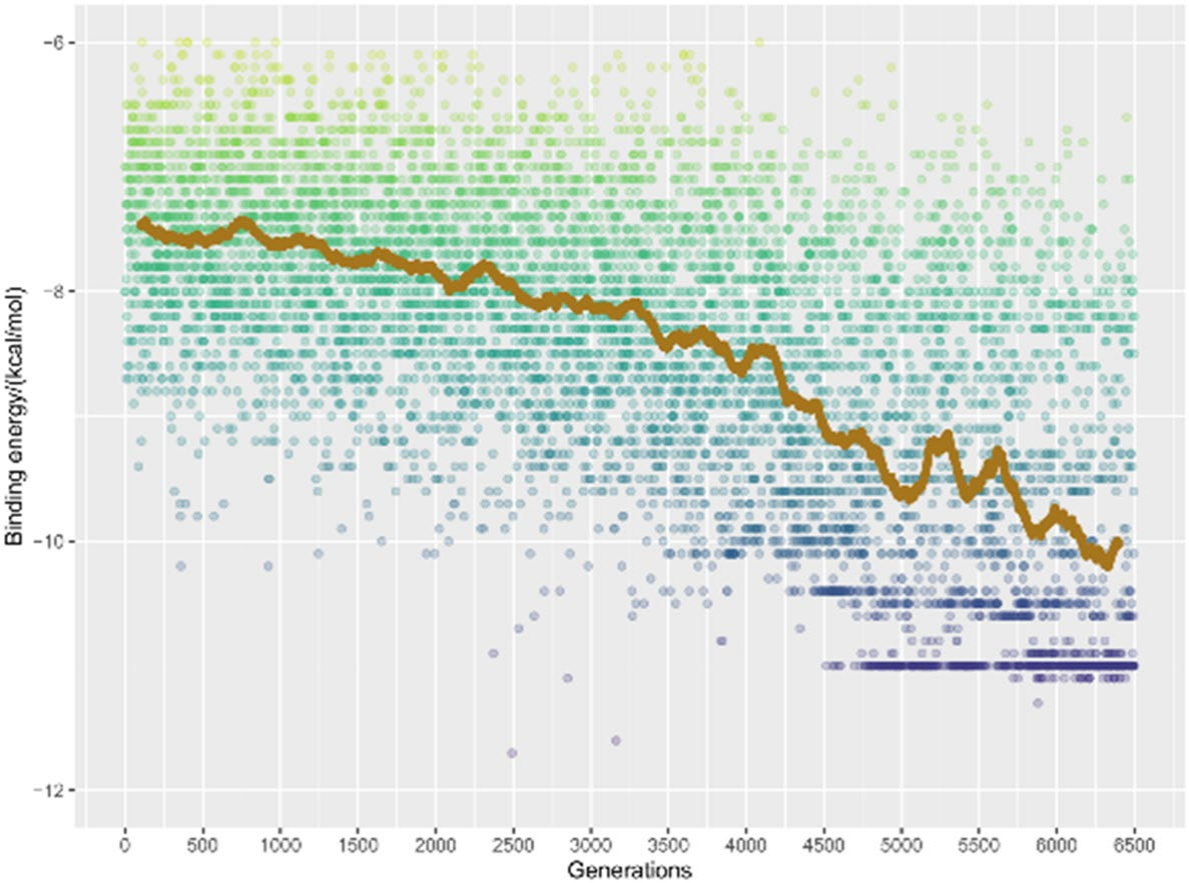
The binding energy between the generated moiety and Aβ receptor (4TVK), the brown line indicates the moving average over every 200 points.

As mentioned in the simulation details, three features are utilized as incentives in the reinforcement learning procedure, i.e., binding energy, SA, and QED score. As the learning progress move forward, newly generated structure should have relatively low binding energy, at the same time, it should have high SA and QED scores. The binding energy of generated structures was shown in Fig. 8. From the averaged value we can see that the binding energy decreases with increasing number of generated structures. The binding energy is seen to range from − 6 to − 12 kcal/mol. The binding energy for model structure is − 6.7 kcal/mol, which suggests that most of the generated structures have preferable affinity toward Aβ receptor. Such result suggests that the implementation of the reinforcement learning is successful, at least it meets the characteristic of binding energy.

**Figure 9:**
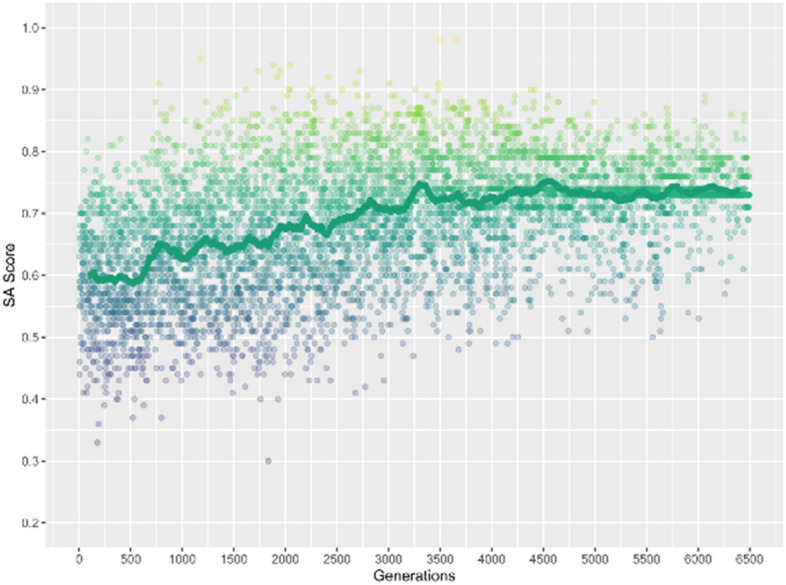
The SA score for all the generated structures based on the model structure using reinforcement learning simulation, the brown line indicates the moving average over every 200 points.

In Fig. 9 the SA score as a function of the number of generated structures has been shown. It can be seen that the SA score first increases with increasing of generated structures, after around 4500 generations, the SA score converges at 0.74. In contrast, the SA score for the model structure of the reinforcement learning is 0.95. Hence, it seems that all generated structures have lower SA score than that of the model structure. This can be rationalized by that the model structure is a well-known moiety, and all the generated structures are the derivatives of the model structure. Nevertheless, the increased and converged trend of SA score suggest that the implementation of the reinforcement learning meets the second characteristics.

**Figure 10:**
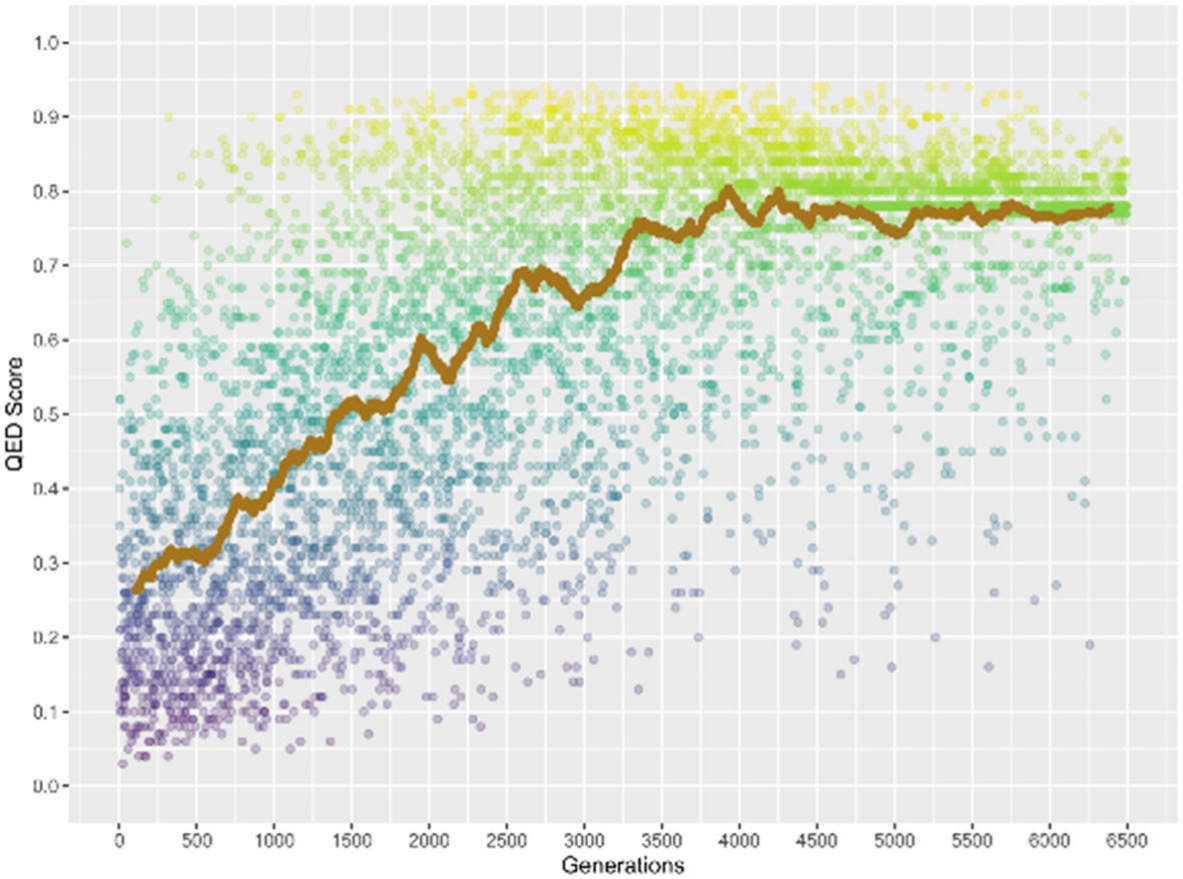
The QED score for all the generated structures based on the model structure using reinforcement learning simulation, the brown line indicates the moving average over every 200 points.

In Fig. 10 the QED score as a function of the number of generated structures has been shown. Similar to the results that were seen in SA score, it can be seen that the QED score first increases with rising of generated structures, the QED score converges around 0.78 after 4000 generations. Moreover, it can be seen that only few generated structures could have a QED score higher than that of the model structure, which is 0.88, and a considerable portion of the generated structures only have a QED score around 0.78. Thus, combining the above results of binding energy and SA score, the results of QED score suggest that all three characteristics have been satisfied.

**Scheme 2:**
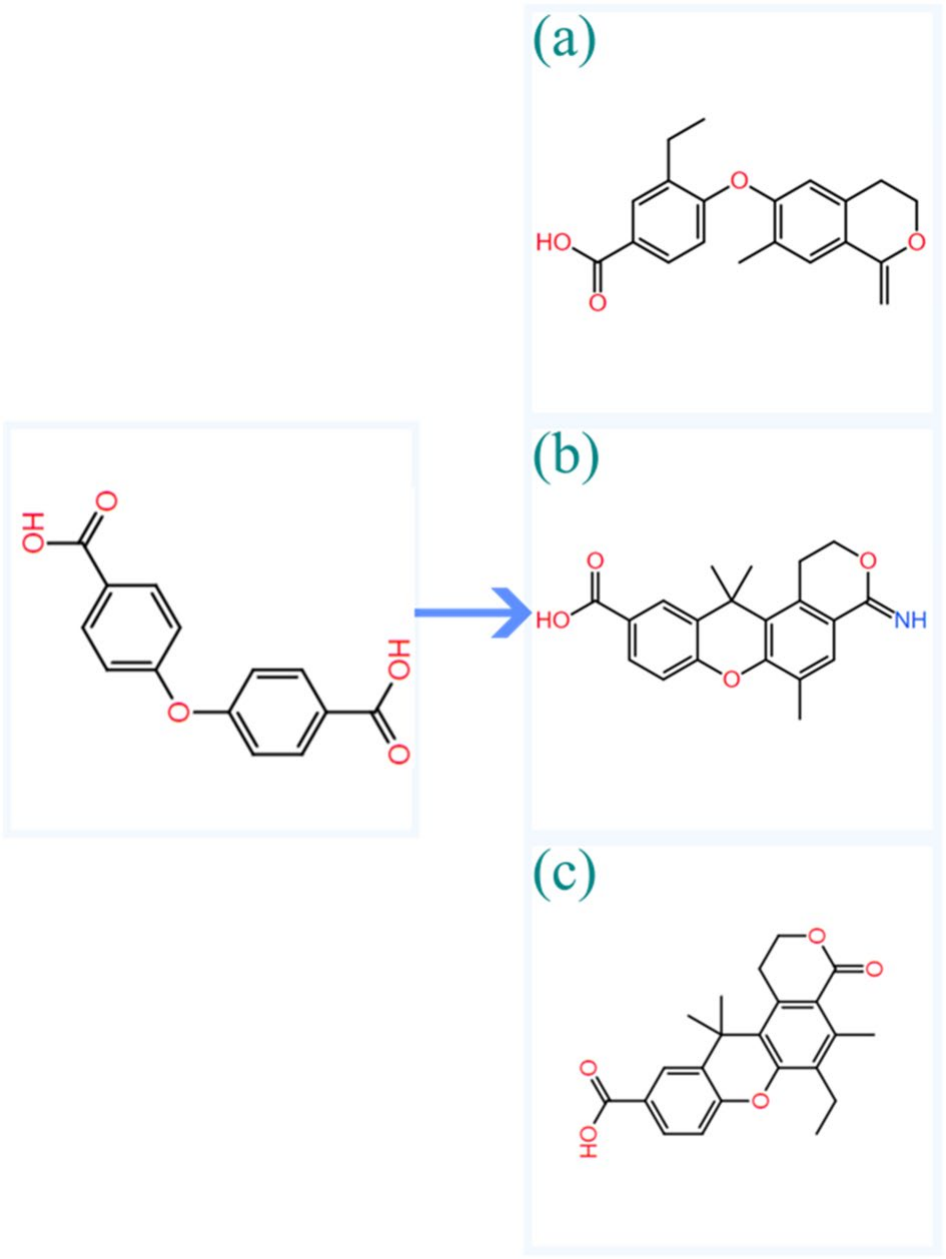
Model structures utilized as inputs in the reinforcement learning simulation (left panel) and three selected structures that satisfy all three thresholds (right panel).

The above results have demonstrated that the implementation of the reinforcement learning simulation meets the characteristics of binding energy, SA score and QED score. Based on the above converged values for SA and QED scores, 0.78 and 0.80 are set as the thresholds for SA and QED scores, these thresholds conduct 25.5% and 25.7% of generated structures that would have higher SA and QED scores. Further, the threshold for binding energy is set to − 10.0 kcal/mol, which conducts 18.4% of generated structures have lower binding energy than the threshold value. Three representative structures were choosing from the structural reservoir in which an effective structure should satisfy all three thresholds. These structures are shown in Scheme 2. Their binding energies are − 10.2, − 10.5 and − 10.6 kcal/mol, their SA scores are 0.82, 0.77 and 0.76, and the QED scores are 0.89, 0.82 and 0.80.

**Figure 11:**
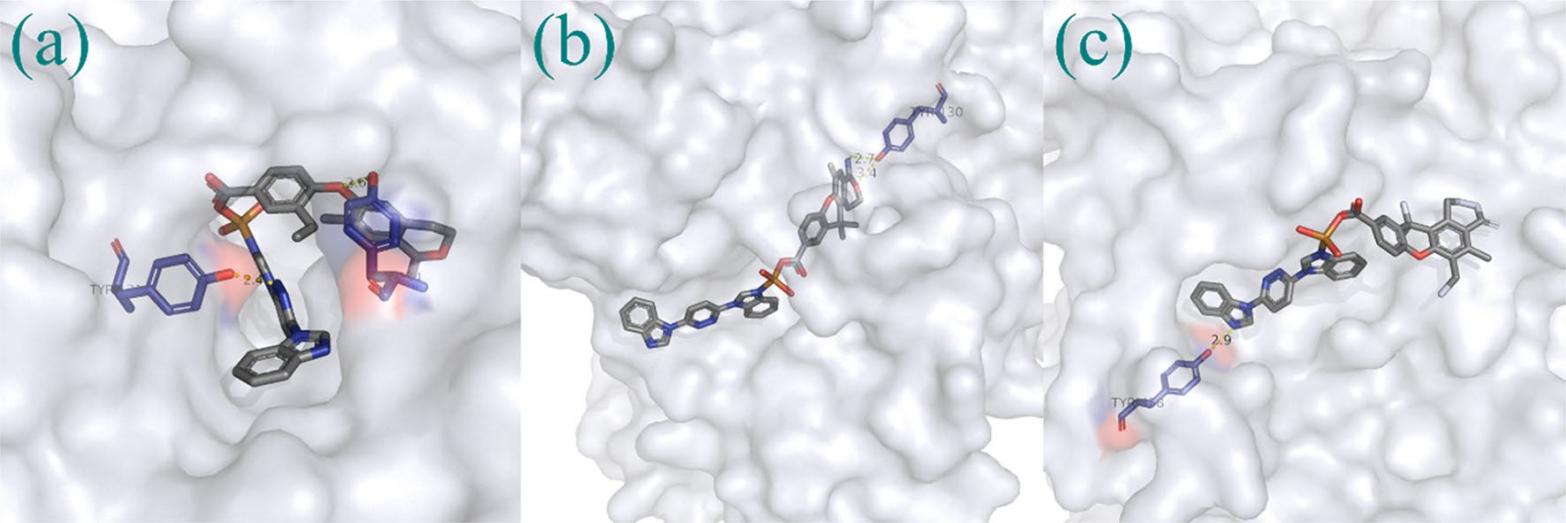
The binding poses between the generated structures that were shown in Scheme 1 and the Aβ receptor (4TVK), their binding energies and inhibition constants are (**a**): -10.27 kcal/mol and 29.85 nM; (**b**) -10.79 kcal/mol and 12.39 nM; and (**c**): -11.17 kcal/mol and 6.44 nM.

To investigate the biological activity of the generated structures with Cu ions and co-ligands more, we carried out molecular docking simulations. Their binding positions are shown in Fig. 11. As indicated in Fig. 11a, the interaction forms are very similar to that was observed in Fig. 7, meaning the pyrimidine and ether groups are the hydrogen bond donors, and the phenolic hydroxyl groups on residues TYR-121 (2.4 Å) and TYR-334 (2.6 Å) are the acceptors of the hydrogen bonds. In Fig. 11b it can be observed that two hydrogen bonds are formed with active residue TYR-130, the oxygen atom on the pyrimidine group is the donor for the first hydrogen bond (3.4 Å), and the hydrogen on the imino group that is connected with pyrimidine group is the acceptor for the second hydrogen bond (2.7 Å). In Fig. 11c only one binding interaction has been formed between imidazole and phenolic hydroxyl groups on residue TYR-458, the corresponding hydrogen bond length is 2.9 Å. Further, the binding energies for these binding poses are − 10.27, − 10.79 and − 11.17 kcal/mol. The results suggest that all three generated structures have comparable biological activity based on their binding energies and hydrogen bond lengths when comparing to the model structure, and the results shown that the reinforcement learning simulation is a powerful method for generating and producing new drug molecules with biological activity.

## Conclusion

In conclusion, we have successfully achieved a new Cu(II)-based coordination polymer via reaction of Cu(NO_3_)_2_·3H_2_O with the 3,6-bis(benzimidazol-1-yl)pyridazine ligand in existence of a V-shape carboxylic acid co-ligand. The structural and chemical components were examined via single-crystal XRD, IR spectra and elemental analysis. Based on the chemical synthesis method, we successfully prepared HA/CMCS hydrogels and studied their micromorphology by scanning electron microscopy. This hydrogel fully inherits the excellent biocompatibility of natural polysaccharides and exhibits a unique three-dimensional porous network structure. Its internal pores are highly interconnected, and the pore size is mainly concentrated in the range of 195.41 ± 3.94 μm, which provides an ideal place for the loading and release of drug molecules. Using paclitaxel as a drug model, we further synthesized a new HA/CMCS-CP**1**@Paclitaxel. Our results suggest thatHA/CMCS-CP**1**@Paclitaxel inhibit the proliferation of thyroid cancer by regulating the expression of S100A6 and ARID1A. This system is expected to be developed as a novel drug for the treatment of thyroid cancer.

The molecular docking simulation results confirmed that the Cu complexes had good biological activities. Further, using Cu complex as the model structure, reinforcement learning has been performed for developing novel drug molecules, the results show that the optimized structures have comparable biological activity based on their binding energies and hydrogen bond lengths when comparing to the model structure, and the results further shown that the reinforcement learning simulation is an useful method for gaining new drug molecules with biological activity.

## Data Availability

Supporting data derived from the results of this research are obtainable upon contact with the corresponding author.
